# Editorial: Omics Approaches in Aquatic Nutritional Physiology

**DOI:** 10.3389/fphys.2022.870482

**Published:** 2022-03-03

**Authors:** Bruno C. Araújo, Qingheng Wang, Chuangye Yang, Erchao Li

**Affiliations:** ^1^Cawthron Institute, Nelson, New Zealand; ^2^Fisheries College, Guangdong Ocean University, Zhanjiang, China; ^3^Department of Aquaculture, College of Marin Sciences, Hainan University, Haikou, China

**Keywords:** physiology, aquaculture, nutrition, nutrients, molecular tools

Diet composition greatly affects animal performance and quality by modulating energetic metabolism, and alterations in key metabolic pathways cannot be frequently characterized using traditional methodologies. After ingesting, digesting, and absorbing feed, nutrients and feed additives influence gene transcription of metabolic pathways, resulting in the expression of several proteins, activating enzymes, and synthesizing innumerable metabolites (Martin and Krol, 2017). Therefore, different nutrients at different levels result in differential performance and composition, and these changes can be successfully investigated by the gene expression profile and consequently by the expression, synthesis and activation of different proteins and metabolites. Thus, omics approaches, including genomics, metagenomics, transcriptomics, proteomics, metabolomics, and lipidomics, are considered important tools in “modern” nutritional studies, providing a holistic overview of how diet composition modulates metabolism, consequently influencing performance, and quality (Martin and Krol, 2017).

This Research Topic aimed to publish manuscripts that highlight new developments in the broad application of omics approaches toward aquatic nutritional physiology. In total, six high-quality manuscripts were published. In an experiment performed with coral trout (*Plectropomus leopardus*) juveniles, Wei et al. used transcriptome analysis to build a transcript database and showed that dietary trivalent chromium [Cr(III)] exposure upregulated the expression of several lipid-relevant genes, consequently modulating lipid metabolism and affecting steroid and terpenoid backbone biosynthesis pathways. The results observed in this study showed that a high dose of organic dietary Cr(III) exposure resulted in stress and sublethal toxic effects in this important commercial species. The authors suggested that a cautious health risk assessment of dietary Cr(III) intake is highly recommended in natural and commercial diets for marine fish, which has previously been largely ignored.

In another study using transcriptome sequencing, Hou et al. evaluated the growth performance, immunity, and transcriptome response of white shrimp (*Penaeus vannamei*) fed the dietary Wnt/β-catenin pathway activator TWS119 (an important immunostimulant for shrimp species). The results showed that shrimp fed with TWS119 at a dose of 16 mg/kg^−1^ significantly improved growth and immunity, and these results can be mainly correlated to the modulation of ribosome function and energy metabolism. The promising results observed in this study provide new insights for immunostimulant exploitation in aquaculture as regulators of critical immune signaling pathways.

Peanut worm (*Sipunculus nudus*) is an important economic annelid species in China, especially due to its high nutritional and medicinal value (Li et al., 2017; Huang et al.). However, nutritional studies with this species are scarce. Thus, to improve production, it is essential to investigate the influence of diets with different macronutrient levels on metabolism and performance. Therefore, Huang et al. investigated the influence of two diets with different carbohydrate and protein levels on the growth of peanut worm juveniles. In addition, the metabolomic responses in animals fed different experimental diets were compared. The results indicated a better performance in animals fed diet containing 35% carbohydrate and 24% protein based on survival, weight gain, and specific growth rate. Additionally, it was revealed a total of 83 significantly different metabolites (SDMs) between animals fed different experimental diets. The results of this study are important for understanding the effects of diet composition on the performance of this promising aquaculture species.

Lipids (and fatty acids, FA), along with proteins, are the most important macronutrients for marine organisms. They are essential molecules for energy production in addition to playing important roles in reproduction, eicosanoid synthesis and as a compound of membranes during development (Tocher et al., 2008). For these reasons, it is essential to investigate the influence of dietary lipids and FA on performance and metabolism of aquatic animals. In an experiment performed with withe shrimp (mysis stage), Visudtiphole et al. evaluated growth and lipidomics responses by supplementation with *Aurantiochytrium limacinum*, a single-cell protist rich in lipids and omega 3 long-chain polyunsaturated FA (n3 LC-PUFA). Despite the importance of n−3 LC-PUFAs in development, no differences in performance were observed in larvae fed the *A. limacinum* diet compared to the control (fed only with Artemia). However, a differential retention of n−3 LC-PUFAs was observed, providing evidence of the importance of these FAs in the advanced stages of this species.

Historically, fish oil (FO) is the main lipid source used in aquafeeds, mainly due to the presence of high n−3 LC-PUFA levels (Glencross, 2009; Turchini et al., 2009). However, the requirement for these FAs to marine organisms is very specific, highly varying according to life stage. Thus, it is important to evaluate the inclusion of n−3 LC-PUFA sources not only in the initial phases but also in adult animals and broodstocks. Using liver transcriptome analysis, Wang et al. evaluated the effects of dietary FO on the gene expression pattern of adult female spotted scat (*Scatophagus argus*). The results showed that FO diets resulted in a significant increase in n−3 LC-PUFA deposition in the ovary, liver, and serum and influenced higher production of serum vitellogenin. Transcription analysis showed that 764 genes were differentially expressed in the liver of fish fed FO compared to those fed diets with soybean oil, corroborating the hypothesis that the physiological response in fish liver is strictly associated with dietary lipids. As previously mentioned, FO is an important lipid source in marine aquafeeds. However, during its production, storage, and usage, this ingredient is readily oxidized, producing a variety of harmful primary and secondary metabolites (Yu et al., 2021). Therefore, Long et al. investigated the effects of the inclusion of oxidized FO on spleen index, antioxidant activity, and transcriptome in hybrid grouper (♀ *Epinephelus fuscoguttatus* × ♂ *Epinephelus lanceolatus*) juveniles. The results showed that oxidized FO resulted in a differential spleen transcriptome profile, oxidative stress, and consequently tissue damage. These results pointed out the importance of assessing FO integrity prior to their inclusion in aquafeeds.

The manuscripts presented in this Research Topic showed a great potential to use omics approaches in the understanding of physiologic processes. As the technology becomes more cost-effective it is expected that omics tools play an important role in the optimization of processes, especially related to precision nutrition of the main aquaculture species.

## Author Contributions

BA, QW, CY, and EL: conceptualization and writing. All authors contributed to the article and approved the submitted version.

## Conflict of Interest

The authors declare that the research was conducted in the absence of any commercial or financial relationships that could be construed as a potential conflict of interest.

## Publisher's Note

All claims expressed in this article are solely those of the authors and do not necessarily represent those of their affiliated organizations, or those of the publisher, the editors and the reviewers. Any product that may be evaluated in this article, or claim that may be made by its manufacturer, is not guaranteed or endorsed by the publisher.
